# Brain activation during phonological and semantic processing of Chinese characters in deaf signers

**DOI:** 10.3389/fnhum.2014.00211

**Published:** 2014-04-16

**Authors:** Yanyan Li, Danling Peng, Li Liu, James R. Booth, Guosheng Ding

**Affiliations:** ^1^State Key Laboratory of Cognitive Neuroscience and Learning, Beijing Normal UniversityBeijing, China; ^2^Department of Communication Sciences and Disorders, Northwestern UniversityEvanston, IL, USA; ^3^Center for Collaboration and Innovation in Brain and Learning Sciences, Beijing Normal UniversityBeijing, China

**Keywords:** congenitally deaf, reading, rhyming, meaning, fMRI

## Abstract

Previous studies found altered brain function in deaf individuals reading alphabetic orthographies. However, it is not known whether similar alterations of brain function are characteristic of non-alphabetic writing systems and whether alterations are specific to certain kinds of lexical tasks. Here we examined differences in brain activation between Chinese congenitally deaf individuals (CD) and hearing controls (HC) during character reading tasks requiring phonological and semantic judgments. For both tasks, we found that CD showed less activation than HC in left inferior frontal gyrus, but greater activation in several right hemisphere regions including inferior frontal gyrus, angular gyrus, and inferior temporal gyrus. Although many group differences were similar across tasks, greater activation in right middle frontal gyrus was more pronounced for the rhyming compared to the meaning task. Finally, within the deaf individuals better performance on the rhyming task was associated with less activation in right inferior parietal lobule and angular gyrus. Our results in Chinese CD are broadly consistent with previous studies in alphabetic languages suggesting greater engagement of inferior frontal gyrus and inferior parietal cortex for reading that is largely independent of task, with the exception of right middle frontal gyrus for phonological processing. The brain behavior correlations potentially indicate that CD that more efficiently use the right hemisphere are better readers.

## Introduction

It is generally believed that there is an intimate connection between language acquisition and subsequent reading development (Perfetti, 1987). Increasing evidence indicates that spoken language skills, especially the child's sensitivity to phonological structures, are fundamental and essential for early and long-term reading acquisition (Dickinson et al., 2006). Correspondingly, one prominent theory argues that reading acquisition builds on the mapping from orthography to phonology, and that a word's meaning will become accessible via the existing phonology-to-semantics link in the speech system (Chall, 1967; Perfetti, 1987). Thus, the children's phonological sensitivities play a pivotal role in the reading development (Temple et al., 2003; Vellutino et al., 2004).

Congenitally and profoundly deaf children cannot access speech before learning to read. This makes the process of learning to read in deaf individuals distinct from the hearing individuals (Perfetti and Sandak, 2000; Geers, 2003). Hence, one may wonder if the deaf individuals can be aware of phonology and how their phonological representations affect their reading development. As to whether deaf people can be aware of phonology, there is inconsistency in previous studies. About half of the studies have found evidence for phonological coding and awareness in deaf individuals, whereas about half have not (Mayberry et al., 2011). Some deaf individuals are aware of phonology, suggesting that they can obtain phonological knowledge from visual and/or articulatory modalities (Dodd and Hermelin, 1977; Hanson and Fowler, 1987). However, they still perform more poorly than hearing individuals on a variety of phonological tasks (Hanson and Fowler, 1987; Campbell and Wright, 1988; Sterne and Goswami, 2000; Aparicio et al., 2007; MacSweeney et al., 2008).

Deaf individuals also seem to have difficulty in the semantic knowledge of written words (Ormel et al., 2010). Because deaf readers may not automatically access phonology, semantic knowledge may provide an important source of reading support (Kyle and Harris, 2006). However, most research has suggested that deaf individuals have semantic processing deficits in alphabetic writing systems (Green and Shepherd, 1975; Marschark et al., 2004; Ormel et al., 2010). For example, both hearing and deaf groups showed significant unmasked priming RT effects and N400 effects, whereas only hearing individuals showed a behavioral effect during masked priming (MacSweeney et al., 2004). Thus, when more automatic word processing is required, the impact of language experience or reading level becomes evident. On the other hand, a similar right visual field advantage was found during a semantic task in deaf and hearing individuals (D'Hondt and Leybaert, 2003), and therefore it is possible that semantics is less likely to be affected in deaf individuals.

Functional magnetic resonance imaging (fMRI) studies in alphabetic languages have also found that deaf individuals show an altered reading network (Neville et al., 1998; Aparicio et al., 2007). Specially, Neville et al. (1998) tested congenitally deaf individuals during silent reading of written sentences, and found that deaf individuals show robust activation in bilateral prefrontal areas and inferior parietal lobule. Aparicio et al. (2007) investigated pre-lingually deaf individuals with lexical and rhyming decision tasks to written words, and found greater activations in the opercular part of the left inferior frontal gyrus, left inferior parietal lobule and right inferior frontal gyrus. These authors suggested that deaf individuals might preferentially rely on the rule-based letter-sound mappings to overcome their poorly specified phonological representations of words.

To the best of our knowledge, there is no fMRI study exploring the neural mechanisms of Chinese character reading in deaf individuals, using either phonological or semantic tasks. Chinese characters represent the phonology and semantics of the spoken languages differently from that of the alphabetic words. For example, spoken Chinese is highly homophonic, with a single syllable having many distinct meanings, and the writing system encodes these homophonic syllables in its major graphic units, the characters. Thus, when learning to read, a Chinese child is confronted with the fact that a great number of written characters correspond to the same syllable, and phonological information is insufficient to access semantics of a printed character. In addition, many Chinese characters encode meaning by including a semantic radical. Furthermore, the relationship between writing skills and Chinese reading is stronger than that between phonological awareness and reading (Tan et al., 2005a).

Previous behavioral studies have found that Chinese deaf individuals have poorer reading ability than hearing individuals (Feng, 2000). As to whether Chinese deaf individuals can be aware of phonology, previous studies have found Chinese deaf individuals have reduced phonological ability. For example, the spelling errors of hearing individuals tend to be substitutions having a similar pronunciation but no visual similarity (homophone errors) to the target character. However, few homophone errors were observed in Chinese deaf individuals (Fok and Bellugi, 1986). In addition, deaf individuals failed to show articulatory suppression effects in a digit span task, suggesting that they were not using a speech-based phonological code (Chincotta and Chincotta, 1996).

Neuroimaging studies have revealed a set of cortical regions shared by alphabetic words and logographic Chinese. The common left hemisphere regions include fusiform gyrus, inferior parietal lobule, middle temporal gyrus, and inferior frontal gyrus (Turkeltaub et al., 2002; Jobard et al., 2003; Price et al., 2003; Tan et al., 2005b). Different nodes of this network are thought to be associated with specific cognitive processes in reading and in oral language more generally. The middle portion of fusiform gyrus (close to the inferior temporal gyrus), labeled by some as the visual word form area (VWFA), has been implicated in the computation of orthographic processes (Cohen et al., 2000; Tan et al., 2001; Vinckier et al., 2007). The left inferior parietal lobule seems to play an important role in mapping of written symbols to the phonological representations (Booth et al., 2002a, 2004, 2006; Eden et al., 2004). The left middle temporal gyrus is thought to be involved in representing semantic information (Booth et al., 2002b, 2006). Finally, the left inferior frontal gyrus is thought to be involved in controlled retrieval and selection, with the dorsal portion (opercular and triangular parts) being involved in phonology (Fiez et al., 1999; Poldrack et al., 1999; Cao et al., 2010) and the ventral portion (orbital parts) being involved in semantics (Poldrack et al., 1999; Friederici et al., 2000; Booth et al., 2006).

Logographic Chinese characters markedly differ from alphabetic words in the nature of their orthography and how they represent the phonology of spoken language. These differences seem to be associated with cross-linguistic differences in their neural basis. The specialized regions for Chinese reading appear to include the right ventral occipito-temporal cortex and left middle frontal gyrus (Bolger et al., 2005; Tan et al., 2005b). The greater involvement of right ventral occipito-temporal cortex is presumably reflecting the greater spatial analysis required of Chinese character recognition (Cao et al., 2009). The left middle frontal gyrus is assumed to serve as a long-term storage center for addressed phonology (Tan et al., 2005b). On the other hand, alphabetic writing systems seem to rely more on the posterior portion of left superior temporal gyrus, which appears to be responsible for assembled phonology (Tan et al., 2003; Eden et al., 2004).

Cross-linguistic differences and similarities in the neural bases of reading have also been investigated in developmental studies. Neuroimaging studies on reading alphabetic words have found that learning to read is associated with enhanced involvement of left fusiform gyrus involved in visual word form recognition (Booth et al., 2004; Brem et al., 2006), left inferior parietal lobule involved in orthography-phonology mapping (Bitan et al., 2007; Booth et al., 2007), left middle temporal gyrus involved in semantic processing (Turkeltaub et al., 2003; Chou et al., 2006) and in left inferior frontal gyrus in a variety of tasks (Booth et al., 2001, 2004; Gaillard et al., 2003; Turkeltaub et al., 2003; Szaflarski et al., 2006). In contrast, developmental differences during Chinese character reading is associated with increased activation in right middle occipital gyrus involved in visual-spatial analysis of characters, left inferior parietal lobule involved in phonological processing and left middle frontal gyrus involved in integrating of orthography and phonology (Cao et al., 2009).

In the present study, we explored the neural mechanisms of Chinese character reading in deaf individuals during both phonological and semantic processing tasks. The primary goal was to investigate the extent to which the brain mechanisms involved in reading Chinese characters are determined by early auditory speech experience, so we compared congenitally profoundly deaf to hearing individuals. We adopted a paradigm used in our previous study on children (Cao et al., 2009) and adults (Booth et al., 2006) to examine the neural mechanisms underlying phonological and semantic processing in both group of participants. Based on previous studies, we expected that deaf individuals may show altered recruitment of left-hemisphere language regions and/or increased recruitment of homologs in the right-hemisphere. We also wished to determine whether any of these effects were task specific by examining whether group differences were larger for phonological vs. semantic processing.

## Methods

### Participants

We recruited 27 profoundly congenitally deaf signers (CD, 10 males, mean age 21.3 ± 2.51 years, range 19–28) and 20 hearing controls (HC, 10 males, mean age 21.7 ± 2.20 years, range 19–28). All were right-handed undergraduate or graduate students with no history of neurological or psychiatric illness (except sensorineural hearing loss). All deaf individuals exhibited profound hearing loss (better ear: mean 100.7 ± 8.45 dB, range 91–120; left ear: mean 102.5 ± 8.65 dB, range 91–120; right ear: mean 102.8 ± 8.48 dB, range 91–120). The causes of deafness in all individuals were genetic, pregnancy-related cytomegalovirus or unknown. All deaf individuals had normal intelligence quotient (IQ) scores as determined by Raven's Standard Progressive Matrices (Raven, 1976), as indexed by a higher score than the 50th percentile on the appropriate norms. This test further ensures the deaf participants recruited in our study are not individuals with multiple disabilities, such as mental retardation. None of the deaf individuals wore hearing aids before 6 years old or in the past 3 years. Chinese Sign Language was the primary language of all deaf individuals. Only deaf individuals who got score of 5 on a 6-point scale from each of two experts on Chinese sign language were recruited in our study. Thus, all signers in our study were viewed as experienced signers. Institutional Review Board (IRB) approval was obtained from the State Key Laboratory of Cognitive Neuroscience and Learning at Beijing Normal University and informed written consent was obtained from all participants.

We only included individuals who met all of the following criteria: (1) overall accuracy was more than 70% for either the rhyming or meaning task (2) the ratio of “yes” responses and “no” responses fell into the range of 35–65% for both the rhyming and meaning task to avoid any response bias, and (3) head motion during the fMRI was less than 3 mm in translation or 3° in rotation. Due to these criteria, seven CD were excluded for the rhyming task, and five CD were excluded for the meaning task. After the exclusion, for the rhyming task, there were no significant differences in age [*T*_(38)_ = 0.947, *p* = 0.350] and gender (χ^2^ = 0.921, *p* = 0.337) between the CD and HC. For the meaning task, there were no significant differences in age [*T*_(40)_ = 0.495, *p* = 0.623] and gender (χ^2^ = 1.437, *p* = 0.231) between the CD and HC.

### Experimental procedure

In both the rhyming and meaning tasks, two Chinese characters were sequentially presented in the visual modality and the participants were instructed to determine whether the two characters rhymed during the rhyming task or were semantically related during the meaning task. Each character was presented for 800 ms followed by a 200 ms blank interval. After the second stimulus was removed, a red fixation cross (+) appeared on the screen, indicating the need to make a response during the subsequent interval that jittered between 2200, 2600, and 3000 ms. The participants were asked to press the yes button with their right index finger for matching pairs (rhyming or semantically related) and the no button with their right middle finger for non-matching pairs. Half of the pairs rhymed (or were related in meaning) and half did not. Semantic association strength between the two characters was assessed by 30 Chinese adults using a 7-point scale. They were instructed to judge to what extent the character pairs were related. If the average score was over 4.5 (*M* = 5.95), we considered the pairs semantically related. If an average score was below 3 (*M* = 2.18), we considered the pairs semantically unrelated.

For the rhyming task, four lexical conditions (24 pairs in each) independently manipulated the orthographic and phonological relation between the words in the pair. In two non-conflicting conditions, two words in a pair shared an identical phonetic radical and rhymed (R+P+, for example,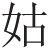/gu1/ and 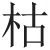/ku1/), or two words in a pair had different phonetic radicals and did not rhyme (R−P−, for example, 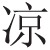 /liang2/ and 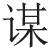/mou2/). In two conflicting conditions, two words in a pair shared an identical phonetic radical but did not rhyme (R+P−, for example, 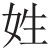/xing4/ and 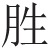/sheng4/), or two words in a pair had different phonetic radicals but rhymed (R−P+, for example, 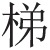 /ti1/ and 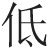/di1/). This manipulation was included to that the rhyming judgment could not be based on orthography alone.

For the meaning task, four lexical conditions (24 pairs each) independently manipulated the orthographic and semantic relation between the words in the pair. In two non-conflicting conditions, two words in a pair shared an identical semantic radical and were related in meaning (R+S+, for example, 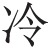/cold/ and 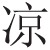/cool/), or two words in a pair had different semantic radicals and were not related in meaning (R−S−, for example, 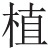/plants/ and 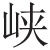/canyon/). In two conflicting conditions, two words in a pair shared an identical semantic radical and were not related in meaning (R+S−, for example, 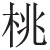/peach/ and 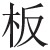/board/), or two words in a pair had different semantic radicals but were related in meaning (R−S+, for example, 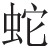/snake/ and 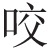/bite/). As with the rhyming task, this manipulation was included so that the relatedness judgment could not be based on orthography alone.

Two types of control trials were used for each task. In the perceptual trials, two unfamiliar Tibetan symbols were presented sequentially in the visual modality. The participants were instructed to press the yes button to identical pairs with their right index finger (for example, 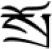 and 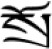) and the no button to different pairs with their right middle finger (for example, 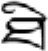 and 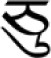). Half of the symbol pairs were identical and half were not. For the fixation trials, participants were instructed to press a button when a black fixation-cross turned blue. The timing for the control trials was the same as for the lexical trials. 24 perceptual trials and 48 fixation trials were used in the each task. The order of lexical and control trials and was optimized for event-related design using OptSeq (http://www.surfer.nmr.mgh.harvard.edu/optseq).

### Stimulus characteristics

Previous studies have found the mean reading age of deaf individuals to be lower than hearing controls (Conrad, 1979; Holt, 1993). To ensure all deaf individuals in our study were familiar with the characters, we selected stimuli from Chinese language textbooks from Grade 1 to Grade 6 in primary schools. The characters (both the first and second words in the pairs) were matched for frequency, age of acquisition (the term when a character is first shown in Chinese language textbooks) (Xing et al., 2004) and strokes across the rhyming and meaning task. In addition, the two characters in the pairs for the rhyming task shared an identical lexical tone, so that this information could not interfere with the rhyming judgment.

### Data collection

Functional and structural images were acquired on a Siemens 3T Tim Trio scanner. Gradient-echo localizer images were acquired to determine the placement of the functional slices. Imaging parameters of reading tasks were: 32 axial slices with an echo-planar imaging (EPI) pulse sequence, repetition time of 2000 ms, echo time of 20 ms, flip angle of 80°, slice thickness of 3 mm, gap of 0.48 mm, FOV = 220 × 206.25 mm, matrix = 128 × 120 × 32; in plane pixel size = 1.71875 × 1.71875 mm. Imaging parameters of the T1-weighted anatomical image were: Sagittal acquisition with a 256 × 256 matrix, repetition time of 2530 ms, echo time of 3.45 ms, flip angle of 7°, number of excitations = 1, 256 mm field of view, 1 mm slice thickness.

### Data analysis

We used SPM5 (http://www.fil.ion.ucl.ac.uk/spm) for preprocessing. The functional images were corrected for differences in slice-acquisition time to the middle slice and were realigned to the first volume in the scanning session. Participants who showed more than 3.0 mm in translation or 3.0° in rotation within a run in any plane were not included. Participants' functional images were co-registered with their own structural MRI images. The co-registered high-resolution structural MRI images were segmented and normalized to the Montreal Neurologic Institute (MNI) template image and spatially re-sampled (2 × 2 × 2 mm). Finally, the images were smoothed with a Gaussian filter of 4 × 4 × 8 mm full width half max (FWHM).

The general linear model was used to estimate condition effects for each participant. There were significant differences in performance between CD and HC in both the rhyming and meaning tasks (see Behavioral Results). To increase the likelihood that the brain differences between the CD and HC were not caused by performance differences, we only included correct items in all analyses. Because this resulted in unequal numbers of items across groups, items were randomly eliminated from HC so that the number items in each condition were equated across groups. There was an average of 78 and 81 lexical pairs during the rhyming and meaning task, respectively. In addition, the CD took longer than the HC to make their decisions. To reduce the effect of reaction time differences on group differences, we covaried this variable when conducting factorial analyses. Two conditions “lexical” and “fixation” were modeled using a canonical hemodynamic response function (HRF) and the contrast “lexical-fixation” was computed. One-sample *t*-tests were applied to determine differences between the lexical and fixation condition, separately for each group and separately for each task. Two-sample *t*-tests were computed separately for the rhyming minus fixation, meaning minus fixation, rhyming minus meaning, and meaning minus rhyming contrasts between groups, with reaction time of lexical judgment as a covariate. We then created a mask of the regions showing group differences either in the rhyming or meaning tasks and the regions showing a group difference in the task effect. Using this mask, we examined positive and negative correlations of accuracy (rhyming/meaning) with signal intensity separately for each task in the CD. We did not examine correlations in the HC as their accuracy was near ceiling. All the reported regions of activation were at *p* < 0.05 AlphaSim corrected (*p* < 0.005 voxel-level cut-off).

## Results

### Behavioral results

In order to determine if there were group differences in the reading and fixation conditions, group (CD vs. HC) by task (rhyming vs. fixation, meaning vs. fixation, or rhyming vs. meaning) ANOVAs were calculated separately on RT and accuracy (Table 1). Significant differences between the HC and CD were found for RT and accuracy in the reading and fixation conditions [RT: rhyming vs. fixation, *F*_(1, 38)_ = 11.310, *p* < 0.01; meaning vs. fixation, *F*_(1, 40)_ = 10.862, *p* < 0.01; accuracy: rhyming vs. fixation, *F*_(1, 38)_ = 50.887, *p* < 0.01; meaning vs. fixation, *F*_(1, 40) = 11.660_, *p* < 0.01]. Significant task effects were found for RT [rhyming vs. fixation, *F*_(1, 38)_ = 139.641, *p* < 0.01; meaning vs. fixation, *F*_(1, 40)_ = 134.749, *p* < 0.01] and accuracy [rhyming vs. fixation, *F*_(1, 38)_ = 139.953, *p* < 0.01; meaning vs. fixation, *F*_(1, 40)_ = 62.637, *p* < 0.01]. Interactions were only found for accuracy [rhyming vs. fixation, *F*_(1, 38) = 47.623_, *p* < 0.01; meaning vs. fixation, *F*_(1, 40)_ = 21.008, *p* < 0.01], but not for RT [rhyming vs. fixation, *F*_(1, 38)_ = 0.897, *p* = 0.350; meaning vs. fixation, *F*_(1, 38)_ = 0.647, *p* = 0.426]. These results indicated that CD showed similarly poor performance in RT in the reading and fixation conditions. Thus, the difference in RT in the reading task between CD and HC are likely due to the different motor plans and/or decision criteria. For reading task, significant group effects were found on RT [rhyming vs. meaning, *F*_(1, 34)_ = 5.950, *p* < 0.05] and accuracy [rhyming vs. meaning, *F*_(1, 34)_ = 53.184, *p* < 0.01]. Significant task effects were found for RT [rhyming vs. meaning, *F*_(1, 34) = 41.558_, *p* < 0.01], but not accuracy [rhyming vs. meaning, *F*_(1, 34) = 2.060_, *p* = 0.160]. No interaction was found for either RT [rhyming vs. meaning, *F*_(1, 34)_ = 0.098, *p* = 0.756] or accuracy [rhyming vs. meaning, *F*_(1, 34)_ = 3.336, *p* = 0.077]. These results indicated that CD showed similarly poor performance in both the phonology and semantics. The group differences in the different lexical conditions are shown in Supplementary Results and Supplementary Table 1.

**Table 1:** **Means (M) and standard deviations (SD) for reaction time (RT in ms) and accuracy (%) in the Rhyming and Meaning tasks for congenitally deaf individuals (CD) and hearing controls (HC)**.

**Session**	**RT**		**Accuracy**	
	**Fixation**	**Reading**	**Fixation**	**Reading**
**RHYMING**				
HC	768 ± 187	1172 ± 231	99.0 ± 1.8	94.5 ± 2.6
CD	1001 ± 268	1345 ± 161	98.8 ± 2.9	81.7 ± 6.9
**MEANING**				
HC	734 ± 211	1035 ± 202	97.0 ± 6.9	94.2 ± 3.8
CD	998 ± 315	1260 ± 260	94.6 ± 6.9	84.3 ± 7.2

### fMRI activation results

**Figure 1:**
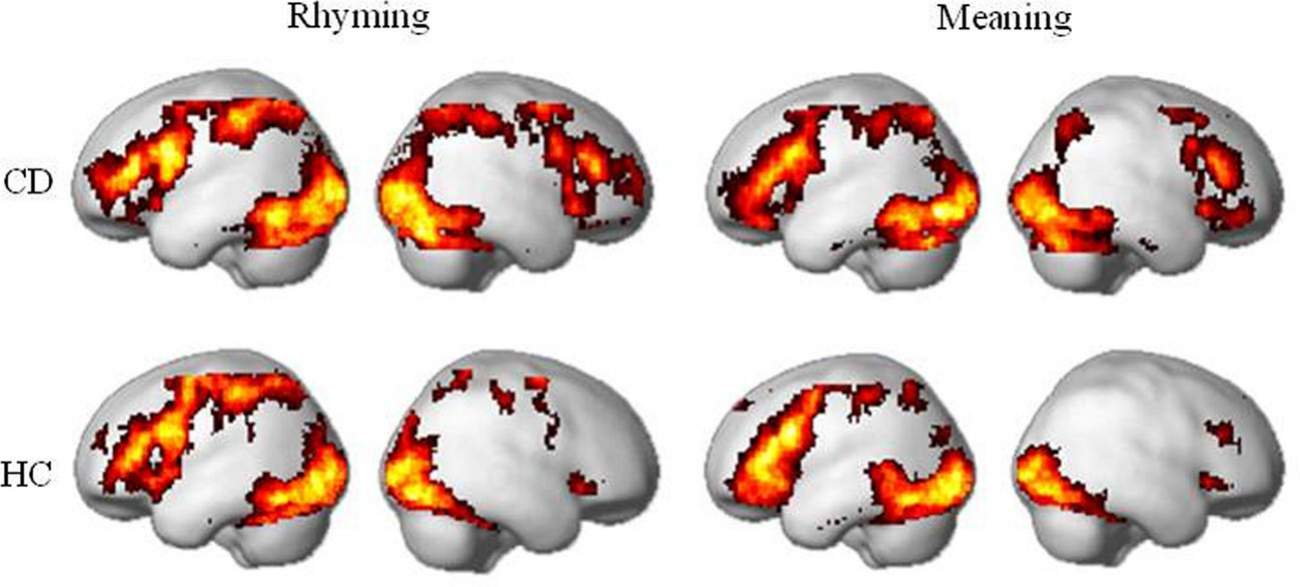
**Brain activity within congentially deaf individuals (CD) and hearing controls (HC) in the rhyming task and the meaning task**. For both tasks, congenitally deaf individuals show similar activity to the hearing controls in the left hemisphere, but enhanced activity in right inferior frontal and inferior parietal cortex. Maps presented at *p* < 0.05 AlphaSim corrected (*p* < 0.005 voxel-level cut-off). CD: congenitally deaf signers; HC: hearing controls.

For both rhyming and meaning tasks, CD and HC showed activation in the reading network including bilateral ventral occipito-temporal cortex (e.g., inferior occipital gyrus and fusiform gyrus), left inferior parietal cortex, left inferior/middle frontal gyrus, and basal ganglia (e.g., putamen and caudate nucleus). These patterns were compatible with previous studies (Tan et al., 2005b; Booth et al., 2006; Chou et al., 2009; Cao et al., 2010). CD appeared to show greater activation in the right inferior parietal cortex and right inferior frontal gyrus. The results were shown in Figure 1.

**Figure 2:**
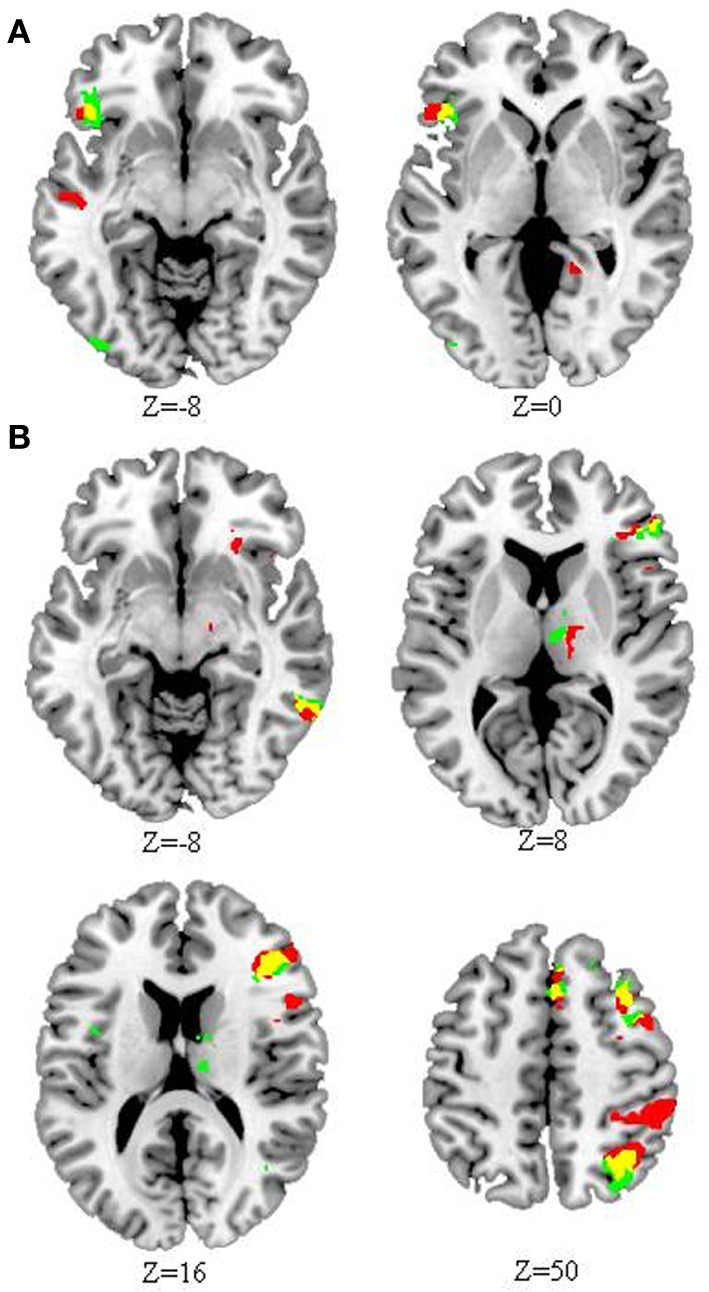
**Differential activation in congenitally deaf (CD) compared to hearing control individuals (HC) within the rhyming and meaning tasks**. **(A)** Reduced activation in CD compared to HC in the rhyming (red) and meaning (green) tasks. **(B)** Greater activation in CD compared to HC in the rhyming (red) and meaning (green) tasks. For both **(A)** and **(B)**, yellow indicates overlap. Compared to HC, CD showed reduced activation in left inferior frontal cortex, but greater activation in right inferior frontal, inferior parietal, and inferior temporal cortex, among other regions, for both tasks. The threshold for the whole brain comparisons was set at *p* < 0.05 AlphaSim corrected (*p* < 0.005 voxel-level cut-off). The number below each map (Z) represents axial coordinates in MNI space.

Compared to HC, CD showed less activation in the left inferior frontal gyrus, but greater activation in right hemispheric regions for both the rhyming and meaning tasks, including the triangular part of inferior frontal gyrus, middle frontal gyrus, angular gyrus, inferior temporal gyrus, cingulate gyrus, thalamus, and superior frontal gyrus (Figure 2). For the rhyming task, CD also showed less activation in left middle temporal gyrus and right precuneus, but greater activation in right orbital part of inferior frontal gyrus, inferior parietal lobule, and supramarginal gyrus. For the meaning task, CD showed less activation in right inferior occipital gyrus and superior temporal gyrus, but greater activation in right insula (Tables 2, 3, Figure 2). We also compared the group differences before partialing for RT. The result is shown in the Supplementary Figure 1 (please see the Supplementary Results) which is very similar to that shown in Figure 2.

**Table 2:** **Comparisons between congenitally deaf individuals (CD) and hearing controls (HC) for the rhyming task**.

**Location**	**H**	**BA**	***Z*-value**	**Volume (mm^**3**^)**	**Coordinates**		
					***x***	***y***	***z***
**CD < HC**							
Triangular/orbital inferior frontal gyrus	L	45,47	4.80	1792	−50	24	2
Precuneus/cuneus/lingual gyrus	R	19,17	4.17	680	16	−48	6
Middle temporal gyrus	L	21	3.99	608	−52	−14	−10
**CD > HC**							
Middle frontal gyrus/opercular inferior frontal gyrus	R	9,8,6	4.71	4520	34	20	52
Triangular inferior frontal gyrus/middle frontal gyrus	R	45,46	4.52	3520	46	28	26
Angular gyrus/supramarginal gyrus/superior parietal lobule	R	39,40,7	4.32	2128	40	−56	52
Orbital inferior frontal gyrus/middle frontal gyrus	R	47,11	4.28	640	26	24	−8
Cingulate gyrus	R	24	4.26	544	4	4	30
Inferior/middle temporal gyrus	R	37,20	4.24	1200	58	−52	−10
Superior frontal gyrus/cingulate gyrus	B	8,32	4.15	2128	4	18	58
Opercular/triangular inferior frontal gyrus	R	44,45	4.15	544	52	14	16
Postcentral gyrus/supramarginal gyrus	R	43,3	3.89	480	60	−18	36
Thalamus	R		3.83	616	14	−14	−6
Supramarginal gyrus	L	40	3.68	432	−48	−38	40
Supramarginal gyrus	R	40	3.56	512	60	−44	38

H, hemisphere; L, left; R, right; B, bilateral; BA, Brodmann's Area. All coordinates p < 0.05 AlphaSim corrected (p < 0.005 voxel-level cut-off).

**Table 3:** **Comparisons between congenitally deaf individuals (CD) and hearing controls (HC) for the meaning task**.

**Location**	**H**	**BA**	***Z*-value**	**Volume (mm^**3**^)**	**Coordinates**		
					***x***	***y***	***z***
**CD < HC**							
Triangular/orbital inferior frontal gyrus	L	45,47	4.20	2040	−42	22	−6
Inferior/middle occipital gyrus	L	18,19	3.75	440	−42	−82	−8
Superior/middle frontal gyrus	L	10	3.55	472	−22	52	18
**CD > HC**							
Angular gyrus/supramarginal gyrus/middle−superior occipital gyrus/superior parietal lobule	R	39,40, 19, 7	5.55	6112	36	−64	38
Middle frontal gyrus/opercular inferior frontal gyrus	R	9,8,44	4.54	2296	40	10	40
Triangular inferior frontal gyrus/middle frontal gyrus	R	45,46	4.32	2528	50	34	24
Superior frontal gyrus/cingulate gyrus	B	8,6,32	4.00	968	4	22	48
Cingulate gyrus/caudate nucleus	R	24	3.97	848	12	−2	28
Thalamus	R		3.71	528	6	−12	6
Insula/precentral gyrus	L	13,6	3.65	456	−38	0	18
Superior frontal gyrus	R	8	3.57	640	18	40	42
Middle/inferior temporal gyrus	R	21,37	3.36	704	62	−50	−8

H, hemisphere; L, left; R, right; B, bilateral; BA, Brodmann's Area. All coordinates p < 0.05 AlphaSim corrected (p < 0.005 voxel-level cut-off).

**Figure 3:**
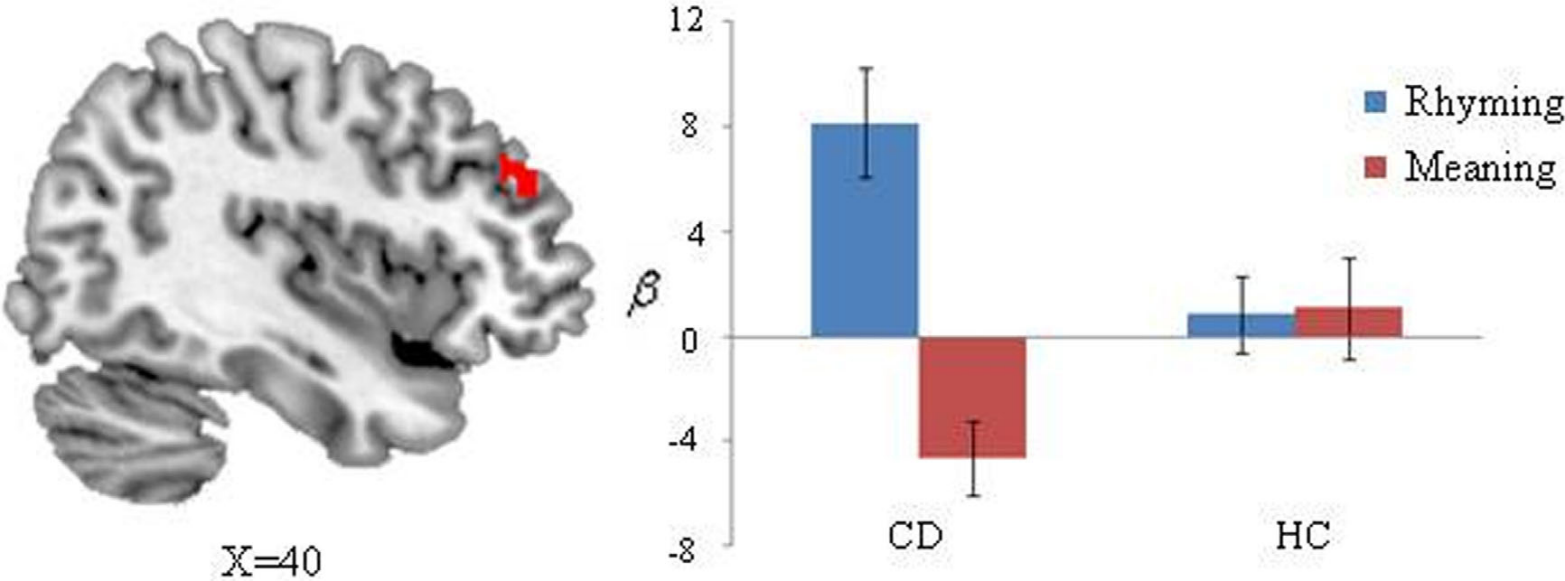
**Task differences selective to the congenitally deaf individuals (CD) compared to hearing controls (HC)**. Bar charts of the right middle frontal gyrus (*x* = 38, *y* = 38, *z* = 20) is plotted for visualization and shows greater activation for the rhyming compared to the meaning task for CD, but no difference for the controls different activations in group contrast in the rhyming compared meaning tasks.

We also found there was greater activation for CD than for HC on rhyming vs. meaning (Figure 3). CD showed greater activation than HC in right middle frontal gyrus (*x* = 38, *y* = 38, *z* = 20) on the rhyming minus meaning contrast. Specially, CD showed greater activation in the rhyming task compared to the meaning task in the right middle frontal gyrus, whereas there were comparable activations across tasks for HC.

When correlating task accuracy with signal intensity during the reading tasks, we found significantly negative correlations between the rhyming task and brain activations. CD who had higher accuracy showed less activation in right angular gyrus and inferior parietal lobule (Table 4).

**Table 4:** **Negative correlations between better reading performance and lower signal intensity within the congenitally deaf individuals for the rhyming task**.

**Location**	**H**	**BA**	***Z*-value**	**Volume (mm^**3**^)**	**Coordinates**		
					***x***	***y***	***z***
Inferior parietal lobule	R	40	4.72	152	30	−40	48
Angular gyrus	R	7	3.39	160	28	−60	40

H, hemisphere; R, right; BA, Brodmann's Area. All coordinates p < 0.05 AlphaSim corrected (p < 0.005 voxel-level cut-off).

## Discussion

To investigate the extent to which the brain mechanisms involved in reading Chinese characters are determined by early auditory speech experience and whether alterations are specific to certain kinds of lexical tasks, we examined the neural mechanisms for the rhyming and meaning judgments of written language in congenitally deaf signers (CD) and hearing controls (HC). Both deaf individuals and hearing controls recruited a left lateralized reading network including ventral occipito-temporal cortex, inferior parietal cortex, and inferior/middle frontal gyrus. This pattern is similar to previous research on hearing Chinese participants (Chou et al., 2009; Cao et al., 2010). Our results are also consistent with previous behavioral studies in alphabetic writing systems by showing that the deaf individuals were less accurate than hearing controls during phonological (Hanson and Fowler, 1987; Campbell and Wright, 1988; Sterne and Goswami, 2000; Aparicio et al., 2007; MacSweeney et al., 2008) and semantic processing (Green and Shepherd, 1975; MacSweeney et al., 2004; Marschark et al., 2004; Ormel et al., 2010). For both tasks, we found that CD showed less activation than HC in left inferior frontal gyrus, but greater activation in several right hemisphere regions including inferior frontal gyrus, angular gyrus, and inferior temporal gyrus. Although many group differences were similar across tasks, greater activation in right middle frontal gyrus was more pronounced for the rhyming compared to the meaning task. Finally, within the deaf individuals better performance on the rhyming task was associated with less activation in right inferior parietal lobule and angular gyrus.

Previous studies have found that learning to read is associated with two patterns of change in brain activation: increased activation in classical left-hemisphere language regions and/or decreased activation in homologous areas in the right-hemisphere (Turkeltaub et al., 2003). Because spoken Chinese is highly homophonic, in learning to read, a Chinese child is confronted with the fact that a great number of written characters correspond to the same syllable. Thus, as children learn Chinese characters, they are required to spend a great deal of time repeatedly copying single characters (Tan et al., 2005a,b). By writing, children learn to decode Chinese characters into a unique pattern of strokes. This orthographic knowledge facilitates the formation of connections among orthographic, phonological, and semantic components of the written Chinese characters (Tan et al., 2005a). When entering elementary school, deaf signers also learn by repeatedly copying characters. Thus, the major difference between the deaf signers and the hearing controls is auditory speech input before learning to read. Due to the lack of early speech experience, CD showed less activation in left hemisphere language regions (i.e., inferior frontal gyrus), whereas they showed greater activation in right hemisphere regions including inferior frontal and inferior parietal cortex during both the rhyming and meaning tasks. CD's engagement of homologous regions of the right hemisphere may be a byproduct of their lack of early speech experience that plays a pivotal role for subsequent learning of written Chinese characters in hearing individuals.

The deaf individuals recruited in the current research primarily used a different language, i.e., Chinese Sign Language, compared to the hearing controls. Increasing evidence shows that reading in deaf people may rely on access to brain networks involved in sign language processing. Behavioral studies have shown that signs were active during written word processing for deaf individuals (Shand, 1982; Morford et al., 2011), and that the sign language translations of written words were activated even when a task did not explicitly require the use of sign language (Morford et al., 2011). Moreover, deaf readers are more likely to become successful readers when they bring a strong sign language foundation to the reading process (Mayberry et al., 2011). Evidences from functional imaging studies found deaf individuals exhibited strong activation not only in left classical language areas but also in right homologous regions including inferior frontal gyrus and/or inferior parietal lobule when processing sign language (Soderfeldt et al., 1997; Bavelier et al., 1998; Neville et al., 1998; Emmorey et al., 2002; Fang and He, 2003; Capek et al., 2008). Taken together, the deaf individuals may rely on sign language mechanisms for skilled reading.

Both CD and HC showed involvement in left triangular/orbital part of inferior frontal gyrus during the rhyming and meaning tasks. Previous studies that suggest the ventral portion of inferior frontal gyrus (orbital and triangular parts) is involved in semantic processing (Poldrack et al., 1999; Friederici et al., 2000; Booth et al., 2006). In addition, compared to hearing individuals, deaf individuals showed decreased activation in this region for both tasks. The reduced activation in ventral inferior frontal gyrus for CD compared to HC may indicate their ineffective retrieval and selection of semantic representations. It is possible that this reduced activation is due to deaf individual's poorer lexical-semantic skills, as show in previous studies (Green and Shepherd, 1975; Marschark et al., 2004; Ormel et al., 2010). However, we did not find that the group difference in left ventral inferior frontal gyrus was larger for the meaning task compared to rhyming task, so future studies are needed to investigate the specific role of ventral inferior frontal gyrus in deaf reading.

We also showed that CD had greater activation than HC in the right triangular part of inferior frontal gyrus for both the rhyming and meaning task. The triangular part of inferior frontal gyrus is thought to be involved in controlled retrieval and selection of phonology (Fiez et al., 1999; Cao et al., 2010). Similar patterns were also shown in previous reading study of deaf individuals (Aparicio et al., 2007). Greater activation in right inferior frontal gyrus may reflect that deaf individuals resort to the right hemisphere for controlled retrieval and selection of phonology. There is another possible interpretation for this compensatory recruitment of right inferior frontal gyrus in deaf individuals. The left inferior frontal gyrus is activated during rhyming judgments, especially for difficult conditions, in hearing individuals (Bitan et al., 2007). Moreover, the activation of inferior frontal gyrus increases with age in hearing individuals, which may be associated with phonological segmentation and covert articulation (Bitan et al., 2007). Therefore, the greater activation in right inferior frontal gyrus during phonological processing in deaf individuals may be due to compensatory recruitment of articulation processes (Aparicio et al., 2007; MacSweeney et al., 2008, 2009). However, we did not show that group differences in the engagement of right inferior frontal gyrus were larger for the rhyming compared to the meaning task, so future studies should examine the specific role of this right inferior frontal cortex in deaf reading.

Additionally, CD showed greater activation than HC in right inferior temporal gyrus for the rhyming and meaning tasks. The left ventral occiptotemporal cortex is involved in the perception of written alphabetic (Cohen et al., 2000; Vinckier et al., 2007) and Chinese words (Bolger et al., 2005; Tan et al., 2005b), while Chinese reading also elicits activation of the right ventral occiptotemporal cortex (Bolger et al., 2005; Tan et al., 2005b). Chinese characters are comprised of strokes packed into square shape, and therefore the character's spatial arrangement requires holistic and visual-spatial processing (Xue et al., 2005), which requires the engagement of right visual cortex (Warschausky et al., 1996). Previous studies have also revealed that deaf individuals show a right hemisphere advantage when judging whether a word corresponds to the sign, whereas hearing controls show a reverse advantage (Ross et al., 1979). Thus, the increased activation in right ventral occiptotemporal cortex may reflect that deaf individuals used more holistic information to accomplish the reading task.

CD also showed greater activation than HC in right angular gyrus and inferior parietal lobule for the rhyming and meaning tasks. Further analysis found that CD who had better performance during the rhyming task showed less activation in right inferior parietal lobule. This result is compatible with previous research in alphabetic word reading which found right inferior frontal gyrus was only activated in less-proficient deaf individuals but not in proficient ones (Corina et al., 2013). The left inferior parietal system is activated during phonological processing of Chinese characters (Tan et al., 2003). This inferior parietal system appears to be involved in temporarily storing phonological information in working memory (Ravizza et al., 2004). Thus, this inferior parietal system may maintain phonological information to accomplish the reading tasks (Tan et al., 2005b). The greater activation in right inferior parietal system may reflect that deaf individuals resort to right hemisphere to temporarily store phonological information to accomplish the reading task, and the brain behavior correlations potentially indicate that CD who more efficiently use the right hemisphere to store phonological information are better readers. In addition, CD also showed greater activation than HC in right inferior parietal lobule for the perceptual tasks (Supplementary Table 2). Thus, the CD might use the right inferior parietal system to temporarily store information to accomplish the corresponding task.

Finally, CD showed greater activation than HC in right middle frontal gyrus (*x* = 38, *y* = 38, *z* = 20, BA9) on the rhyming minus meaning contrast. Specifically, CD showed greater activation in the rhyming task compared to the meaning task in the right middle frontal gyrus, whereas there were comparable activations across tasks for HC. The left middle frontal gyrus is thought to be specialized for Chinese reading (Perfetti et al., 2005; Tan et al., 2005b). This region has been consistently activated during Chinese reading in hearing adults in various tasks (Tan et al., 2001; Chee et al., 2004; Kuo et al., 2004; Booth et al., 2006; Cao et al., 2009). It has been argued that this area is responsible for addressed phonology in Chinese reading (Tan et al., 2005b). It is interesting to note that Chinese dyslexics exhibited less activation in BA9 in the left hemisphere compared to controls (Siok et al., 2004). Consistent with the findings in dyslexia, Cao et al. (2009) found that lower accuracy children showed reduced activation in BA9 in the left hemisphere. However, in the current study, CD showed stronger activation than HC in right middle frontal gyrus for the rhyming as compared with the meaning task. This finding suggests deaf readers may resort to alternative cognitive mechanisms to overcome their deficits in phonological processing. Previous studies have found that the engagement of right hemisphere regions is homotopic to the left language network in left-hemispheric brain lesions and callosal agenesis (Staudt et al., 2002; Riecker et al., 2007). Consequently, deaf readers might recruit right BA9 to integrate visual orthographic information with addressed phonology. Moreover, our finding is compatible with a previous alphabetic study, which found that deaf individuals showed increased right inferior frontal gyrus activation, and interpreted this as reflecting greater demands on grapheme-to-phoneme conversion (Aparicio et al., 2007).

In conclusion, the use of both a rhyming and a meaning task in the current study allowed us to find: (1) CD showed less activation than HC in left inferior frontal gyrus, but greater activation in right inferior frontal gyrus, angular gyrus and inferior temporal gyrus during both the rhyming and meaning tasks, suggesting CD less effectively engage classical language regions in the left hemisphere involved in Chinese character processing; (2) CD showed greater activation than HC in right middle frontal gyrus for the rhyming as compared with the meaning task, suggesting greater recruitment of right hemisphere for phonological processing in CD; and (3) CD who had better performance on the rhyming task showed less activation in right inferior parietal cortex, potentially indicating that CD that more efficiently use the right hemisphere for phonological storage are better readers.

### Conflict of interest statement

The authors declare that the research was conducted in the absence of any commercial or financial relationships that could be construed as a potential conflict of interest.
