# Supplementary material for: Brain activation during phonological and semantic processing of Chinese characters in deaf signers
Source: Front Hum Neurosci. 2014 Apr 16;8:211. doi: 10.3389/fnhum.2014.00211 (PMC3997016; doi:10.3389/fnhum.2014.00211)
Supplement: Supplementary file 1 [file Presentation1.PDF]

Supplementary Results

1. Behavioral result: Group differences in the different lexical conditions.

In order to determine if there were group differences in the different lexical conditions, group by lexical conditions ANOVAs were calculated separately on RT and accuracy in the rhyming and meaning tasks (Supplementary Table 1). There were significant main effects for group showing that CD were less accurate (rhyming, $F_{1,38}=57.479$, $p<0.01$; meaning, $F_{1,40}=29.852$, $p<0.01$), and slower (rhyming, $F_{1,38}=7.643$, $p<0.01$; meaning, $F_{1,40}=10.437$, $p<0.01$) compared to HC. There were significant main effects for lexical conditions showing that participants were more accurate (rhyming, $F_{3,114}=14.796$, $p<0.01$, meaning, $F_{3,120}=3.069$, $p<0.05$), and faster (rhyming, $F_{3,114}=6.674$, $p<0.01$; meaning, $F_{3,120}=25.339$, $p<0.01$) on the non-conflicting conditions (R+P+ and R-P- for the rhyming task; R+S+ and R-S- for the meaning task) than on the conflicting conditions (R+P- and R-P+ for the rhyming task; R+S- and R-S+ for the meaning task). The interactions between group and lexical condition during the rhyming task were found for accuracy ($F_{3,114}=7.951$, $p<0.01$), but not for RT ($F_{3,114}=2.079$, $p=0.107$). Simple effect analysis found that the accuracy difference between conflicting and non-conflicting conditions was larger in CD (all Ps<0.01), but not in HC (all Ps>0.4) for the rhyming task. The interactions between group and lexical condition during the meaning task were found for accuracy ($F_{3,120}=5.856$, $p<0.01$), and RT ($F_{3,120}=2.873$, $p<0.05$). Simple effect analysis for RT found that R-S- was faster than R+S- for HC, but for CD both S+ were faster than R-S-, and R-S+ was faster than R+S- (all Ps<0.05). Simple effect analysis for accuracy found that R+S+ was more accurate than both S- for CD (all Ps<0.01), but not for HC (all Ps>0.7).

Supplementary Table 1. Means (M) and standard deviations (SD) for reaction time (RT in ms) and accuracy (%) in the four lexical conditions during the rhyming and meaning tasks for congenitally deaf individuals (CD) and hearing controls (HC).

Session				
Rhyming	R+P+	R+P-	R-P+	R-P-
HC (RT)	1147±260	1195±213	1198±272	1158±252
CD (RT)	1243±160	1387±196	1399±189	1366±216
HC (%)	94.3±5.5	92.8±6.3	94.3±6.5	96.2±4.7
CD (%)	87.1±8.6	73.9±10.8	77.5±11.8	89.2±7.1
Meaning	R+S+	R+S-	R-S+	R-S-
HC (RT)	975±195	1114±242	1005±206	1050±219
CD (RT)	1171±272	1377±317	1190±251	1352±292
HC (%)	94.1±6.5	94.3±6.8	91.8±8.5	96.4±4.5
CD (%)	91.5±6.8	78.6±17.5	87.5±9.5	79.6±14.7

2. Imaging Results:

Supplementary Table 2. Comparisons between congenitally deaf individuals (CD) and hearing controls (HC) for the perceptual task.

	Volume	Coordinates

Location	H	BA	Z value	(mm^3)	x	y	z
Inferior parietal lobule	R	40	4.57	432	50	-46	40
Inferior parietal lobule	R	7	4.52	160	36	-54	46

Note. H, hemisphere; R, right; BA, Brodmann's Area. All coordinates $p<0.05$ AlphaSim corrected ($p<0.005$ voxel-level cut-off)

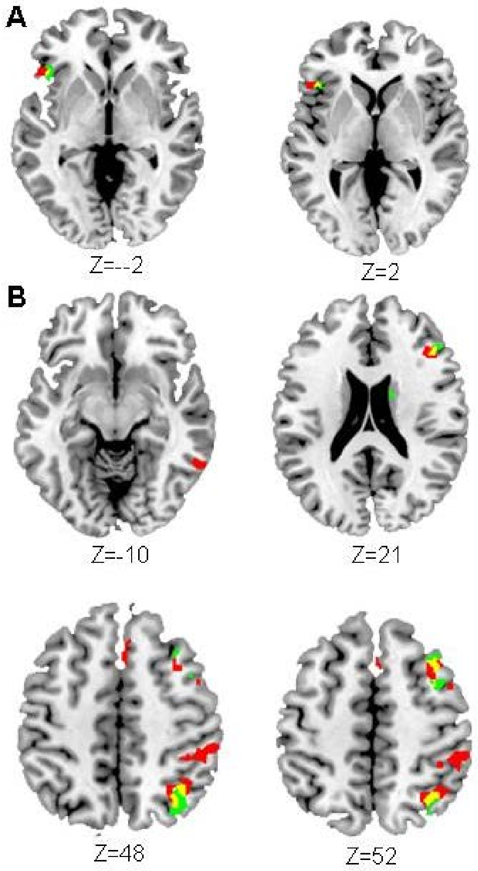

Supplementary Figure 1. The group differences before partialing for RT, which is very similar to the results shown in the main text (Fig. 2). A. Reduced activation in CD compared to HC in the rhyming (red) and meaning (green) tasks. B. Greater activation in CD compared to HC in the rhyming (red) and meaning (green) tasks. The threshold for the whole brain comparisons was set at $p<0.05$ AlphaSim corrected ($p<0.005$ voxel-level cut-off). The number below each map represents axial coordinates in MNI space. CD: congenitally deaf signers; HC: hearing controls.
